# Indoor Positioning System Based on Chest-Mounted IMU

**DOI:** 10.3390/s19020420

**Published:** 2019-01-21

**Authors:** Chuanhua Lu, Hideaki Uchiyama, Diego Thomas, Atsushi Shimada, Rin-ichiro Taniguchi

**Affiliations:** 1 Graduate School of Information Science and Electrical Engineering, Kyushu University, Fukuoka 819-0395, Japan; 2 Library, Kyushu University, Fukuoka 819-0395, Japan; uchiyama@limu.ait.kyushu-u.ac.jp; 3 Faculty of Information Science and Electrical Engineering, Kyushu University, Fukuoka 819-0395, Japan; diego_thomas@limu.ait.kyushu-u.ac.jp (D.T.); atsushi@ait.kyushu-u.ac.jp (A.S.); rin@kyudai.jp (R.-i.T.)

**Keywords:** pedestrian dead reckoning, inertial navigation, accelerometers, gyroscopes, barometers, map matching, particle filters

## Abstract

Demand for indoor navigation systems has been rapidly increasing with regard to location-based services. As a cost-effective choice, inertial measurement unit (IMU)-based pedestrian dead reckoning (PDR) systems have been developed for years because they do not require external devices to be installed in the environment. In this paper, we propose a PDR system based on a chest-mounted IMU as a novel installation position for body-suit-type systems. Since the IMU is mounted on a part of the upper body, the framework of the zero-velocity update cannot be applied because there are no periodical moments of zero velocity. Therefore, we propose a novel regression model for estimating step lengths only with accelerations to correctly compute step displacement by using the IMU data acquired at the chest. In addition, we integrated the idea of an efficient map-matching algorithm based on particle filtering into our system to improve positioning and heading accuracy. Since our system was designed for 3D navigation, which can estimate position in a multifloor building, we used a barometer to update pedestrian altitude, and the components of our map are designed to explicitly represent building-floor information. With our complete PDR system, we were awarded second place in 10 teams for the IPIN 2018 Competition Track 2, achieving a mean error of 5.2 m after the 800 m walking event.

## 1. Introduction

In outdoor positioning systems, GPS and magnetometers are assembled to estimate the position and orientation of a system with respect to Earth’s frame. With a standard GPS on mobile, positioning error can be around 10 m in well-conditioned outdoor environments, such as an open-space area. However, accuracy largely becomes less reliable in indoor environments because not only do the walls of the building block the GPS signals, but also additional magnetic fields from electrical devices produce noises. Therefore, localization using such devices generally works only in outdoor situations.

Nowadays, demand for indoor navigation systems has been rapidly increasing for location-based services, such as navigation to shops, and advertisements. From a technical point of view, indoor navigation systems can be separated into two categories: infrastructure-based systems, and infrastructure-free ones. The first category requires to install devices in the environment, and the position of the pedestrian is computed by using those devices. Radio wave-based systems, such as radio-frequency identification (RFID) [1,2], bluetooth low energy (BLE) [3], and WiFi [4,5], belong in this category. These normally need many resources to calibrate the devices so that all device positions are known. The second category, which does not require any additional hardware to be installed, can be more cost-effective. Camera-based simultaneous localization and mapping (SLAM) and inertial measurement unit (IMU)-based pedestrian dead reckoning (PDR) [6] systems belong in this class, and can provide the relative pose from a starting location. Compared with the first category, they need the initial pose be known by other means, whereas both the sampling rate and the accuracy of the relative pose estimation are quite high. With the recent advances of cameras and SLAM algorithms, SLAM-based navigation systems can work in real time. However, a drawback of camera-based systems is requiring both bright illumination and a static environment, which sometimes cannot be fulfilled in a real environment, such as a shopping mall. Although accuracy is not as high as the SLAM systems, IMU-based PDR systems have no impact on the environment, and have been researched for years.

In the literature of the IMU-based PDR, the IMU can be mounted on different body parts to adapt to various conditions. As the most accurate way, the IMU was mounted on a foot to utilize the framework of the zero-velocity update (ZUPT) [7,8]. The velocity computed by sequentially integrating acceleration normally drifts away from actual value over time due to the accumulation of sensor noise. The ZUPT was proposed to reduce this error as follows. While walking, the velocity of a foot can be considered as zero when the foot is attached on the ground. By detecting these periods and resetting the velocity to zero, the error on the velocity can be suppressed. However, the ZUPT cannot be used for other body parts because there is normally no periodical zero-velocity moment.

A specific motion model was normally proposed according to the body installation position of the IMU. For example, handheld IMU-based systems can be implemented in smartphones [9]. Head-mounted systems can be implemented in smartglasses [10]. Some systems can also recognize where the IMU is mounted and apply different processes according to positions [11,12]. However, it is not easy to accurately compute walking displacement from the IMU attached onto such body parts because there are many unexpected movements in addition to pure walking movement, such as head rotation and hand shaking. Unexpected movements can obviously introduce errors and decrease positioning accuracy.

**Figure 1:**
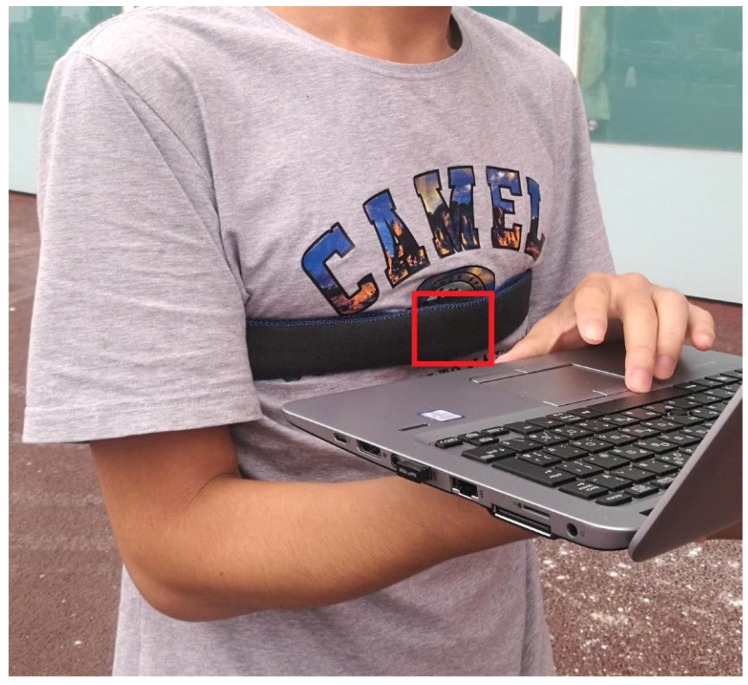
A prototype system of the chest-mounted intertial measurement unit (IMU)-based pedestrian dead reckoning (PDR).

In this paper, we propose an indoor positioning system that uses a chest-mounted IMU with an efficient map-matching algorithm for multifloor navigation tasks. We propose to select the chest as a novel installation position because the chest has the fewest unexpected movements in the upper body while walking. In other words, the chest has lower degrees of freedom for movement compared with other parts, such that it normally faces the moving direction. Therefore, our motivation is to create a body-suit-type PDR system to achieve navigation in a multifloor building. The input data of our system are the temporal sequence of acceleration, angular velocity, and barometric pressure from the IMU, and the floor map for map matching. As a device, we used NGIMU (http://x-io.co.uk/ngimu/), of which the specifications are listed in Table 1, to develop a prototype system, and attached it at the chest with a stretching band, as illustrated in Figure 1. To adapt the PDR system to the chest-mounted IMU, we propose a novel regression model based on step-length estimation. Different from methods for other upper-body-based PDR, our approach can accurately compute not only step length but also step direction. To reduce error accumulation on the position and heading of the pedestrian in the building, we integrated the idea of an efficient map-matching algorithm based on particle filtering into our system. The components of our map were carefully designed, and can explicitly represent building-floor information. As onsite performance evaluation, we were awarded second place in 10 teams for the IPIN 2018 Competition (http://ipin2018.ifsttar.fr/competition/about/) Track 2, achieving a mean error of 5.2 m after walking 800 m.

In summary, the contribution of our paper is threefold as follows.

- A novel chest-mounted IMU-based PDR for body-suit-type systems is proposed.
- Step-length estimation for a chest-mounted IMU is proposed.
- A complete system for multifloor navigation tasks was implemented and the code is open (https://github.com/rairyuu/PDR-with-Map-Matching).

## 2. Related Work

The framework of IMU-based PDR systems generally consists of four parts: orientation update, step detection, step-length estimation, and map matching. Orientation update and map matching are common techniques in navigation systems [13], while step detection and step-length estimation are dedicated to human motion because they use the constraints of a pedestrian step. In this section, we review the existing methods on these three parts.

As the cornerstone of navigation systems, orientation update has been researched for decades. Orientation accuracy heavily affects positioning accuracy. Generally, IMU orientation is computed from gyroscope data. Therefore, its accuracy depends on the accuracy of the gyroscope. In practice, the error on gyroscope data consists of two parts, the high-frequency noise and the slow-changing temperature drift [14]. Many studies have explored methods to reduce noise [15,16]. To suppress the temperature drift, Shen et al. proposed a model based on a genetic algorithm and the Elman neural network [17]. However, the computation cost of removing sensor noise and the temperature drift is relatively high. To achieve a fast orientation update, Madgwick et al. proposed an efficient orientation filter that is robust against the error on sensor data [18]. By utilizing the accelerometer data, the Madgwick filter achieves a high-accuracy orientation update. Moreover, its computation cost is low. Therefore, in our system, we used the Madgwick filter to update the orientation.

The input data for standard IMU-based PDR systems are the temporal sequence of acceleration and angular velocity expressed in the device frame. By computing the gravity direction and using the double integration of the acceleration with the initial velocity after removing gravity acceleration, it is possible to compute the displacement on the 2D map. However, the computed displacement is not accurate over time owing to error accumulation caused by sensor noise. To avoid error accumulation, the process of step detection was proposed. For foot-mounted IMU-based systems, Ojeda et al. proposed two empirical rules to separate steps [7,8]. Madgwick et al. proposed a step-detection algorithm based on filtering and thresholding, which only required the norm of the acceleration [18,19]. For handheld IMU-based systems, step detection is more difficult because hand motion is not always correlated with step motion. To deal with this problem, Susi et al. proposed an adaptive algorithm that first recognized the motion type, then applied a proper method according to the motions [20]. For head-mounted IMU-based systems, a filtering and thresholding-based algorithm is still a good choice, as implemented by Zhang et al. [10].

Step-length estimation is the process of computing the displacement of each step. Displacement consists of two parts: length and direction. This means that this process can be classified into two categories: computing the length and direction, separately or not. The use of a fixed step length computed from, for instance, body height, may work for computing the displacement of normal walking. However, it is generally not accurate for other motions such as running or slow steps. To improve the accuracy, Weinberg et al. proposed an empirical equation to compute the step length with a hip (or upper-body)-mounted IMU [21]. Step direction can be computed from the IMU poses. For foot-mounted IMU-based systems, the double integration of the acceleration can produce both length and direction together. However, this straightforward computation cannot fit all motions. To settle this problem, Shin et al. proposed an adaptive step-length estimation algorithm [22]. For handheld IMU systems, Renaudin et al. proposed an adaptive algorithm based on motion mode classification [23]. Yan et al. proposed a regression model to compute step displacement [11].

Utilizing both step detection and step-length estimation can avoid errors in each step. However, position and heading errors in the global or world frame still accumulate over time. To solve this problem, a map-matching algorithm was proposed by making the constraints of the positions in the global map frame [24,25,26]. The particle filter-based approach is particularly good for dealing with nonlinear and non-Gaussian estimation problems [27]. By implementing the particle filter for a map-matching algorithm, positioning accuracy can be significantly improved. Davidson et al. showed how the particle filter improved the performance of indoor navigation systems [28]. By combining the backtracking particle filter with different levels of a 2D building map, Widyawan et al. achieved highly accurate indoor navigation [29]. Bojja et al. proposed a 3D map-matching algorithm in order to navigate the 3D space [30].

## 3. Proposed System

**Figure 2:**
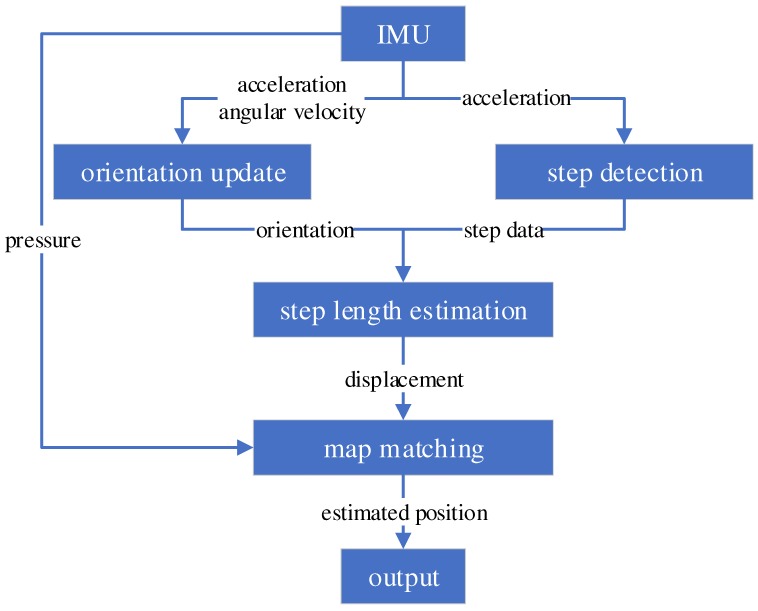
System overview.

As illustrated in Figure 2, our system mainly consists of four parts: orientation update, step detection, step-length estimation, and map matching. This framework is basically the same as the standard used in IMU-based PDR systems. Particularly, we propose novel step-length estimation with a chest-mounted IMU, and we implemented a complete system for multifloor navigation tasks.

In the orientation update, IMU orientation with respect to the world frame, which is the initial IMU sensor frame, was computed. Step detection is the process of separating sequential data into steps. Once one step is detected, the displacement of the step is computed in step-length estimation. Since only the PDR with the IMU contains the positioning and heading errors, consistency between the estimated trajectory and the map is optimized in map matching. In the remainder of this section, we introduce each process in detail.

### 3.1. Orientation Update

**Figure 3:**
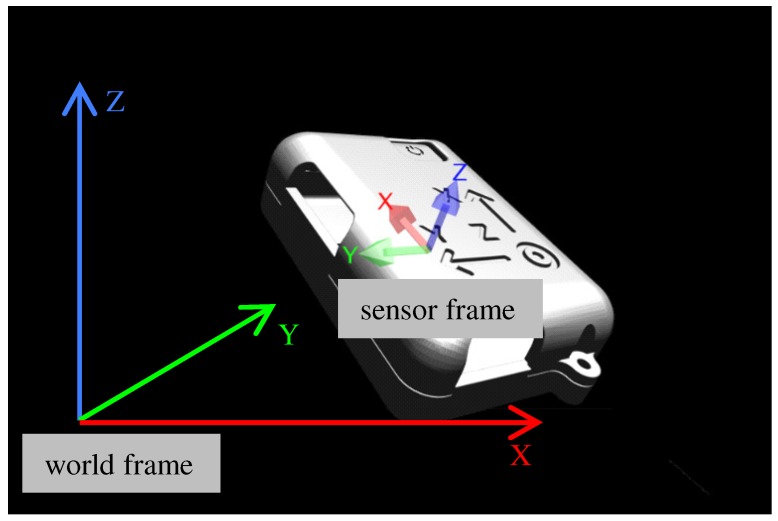
Sensor and world frames.

The data acquired from the IMU are expressed in the IMU sensor frame. To compute the current position in the world frame, we first transform the 3D acceleration data from the sensor frame to the world frame. Normally, we defined the initial IMU sensor frame as the world frame, as illustrated in Figure 3. For instance, the *z* axis can be parallel to the gravity direction when the IMU is placed on the ground at the beginning of the process. In our system, the world frame is defined as follows: the *x* axis is the initial moving direction, the *y* axis is perpendicular to the *x* axis, and the *z* axis is parallel to the gravity direction. The transformation matrix is simply computed with the algorithm by Madgwick et al. [18], which uses Quaternion representation for three degrees-of-freedom (DoF) poses. After this process, we can have acceleration in the world frame to remove gravitational acceleration on the *z* axis, and finally have the acceleration of the device itself.

It should be noted that the orientation computed with the IMU gradually contains errors on the azimuth angle in the world frame, whereas the elevation angle can be compensated with gravity acceleration. For instance, the *x* axis, which is initially defined as the heading, does not match with the actual heading. To compensate this, we use a map-matching algorithm to correct the heading, as explained in Section 3.4. Since we additionally parameterize the correction value against the orientation, we can use any orientation-update method as an external orientation estimator. This means that our system is based on a loosely coupled approach between the PDR and map matching.

### 3.2. Step Detection

**Figure 4:**
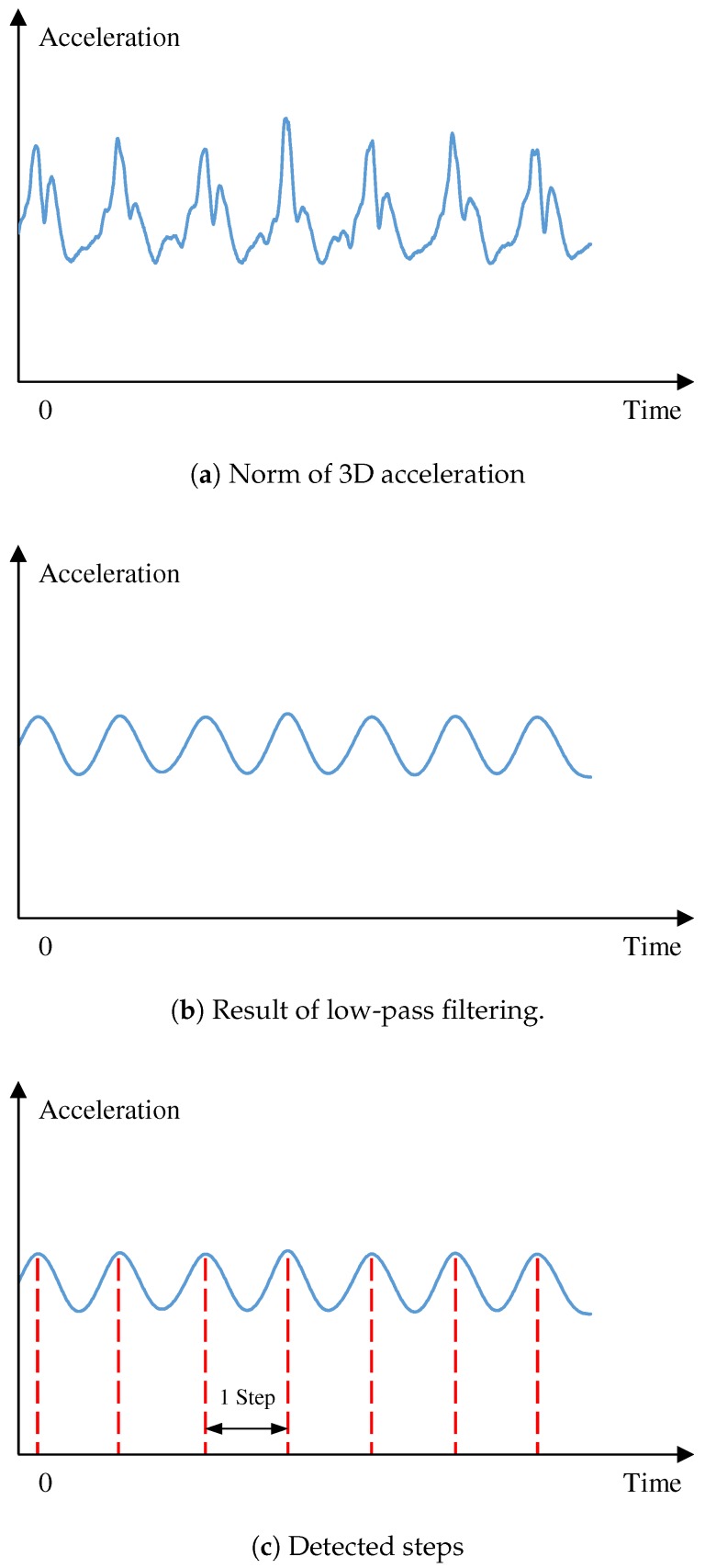
Step-detection process.

To reduce the error accumulation caused by sensor noise, we apply the step-detection method to separate sequential data into steps so that we can independently process the data of each step. As illustrated in Figure 4, we propose to use the filtering and thresholding approach for the acceleration norm to detect steps, similarly to References [18,19] because the peaks in the acceleration-data sequence captured at the chest has similar tendencies as other body parts.

**Figure 5:**
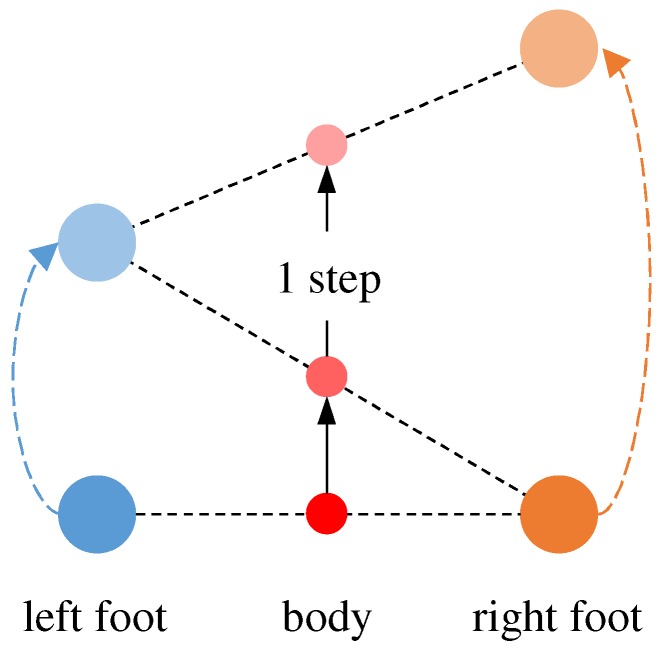
Definition of one step.

First, we compute the 3D acceleration norm in the sequence, as illustrated in Figure 4a. To remove noise, we apply a low-pass filter to the norm sequence. In Figure 4b, the peaks of the result correspond to the moments when the foot hits the ground, where acceleration is maximum. As illustrated in Figure 5, we define one step as the interval from one peak to the next one. An example result of the step detection is illustrated in Figure 4c.

### 3.3. Step-Length Estimation

In the literature of the PDR with an IMU attached on an upper-body part, step-length estimation was achieved by designing an empirical motion model derived from IMU data [21]. Therefore, we followed the same way to derive the motion model for a chest-mounted IMU.

By analyzing the relationship between displacement and the IMU data, we empirically formulated that the displacement of one step can be computed by:

(1) D=K·∫∫a(t)dtdt

where a(t) is the 3D acceleration at time *t*, the range of the integral is the duration of one step, and *K* is a scalar parameter that needs to be calibrated for each user. To avoid drift errors, velocity is not sequentially computed, and Equation (1) takes the initial zero velocity for distance computation. Since the double integration of accelerations is used in our approach, both the length and direction of one step can be computed at the same time.

**Figure 6:**
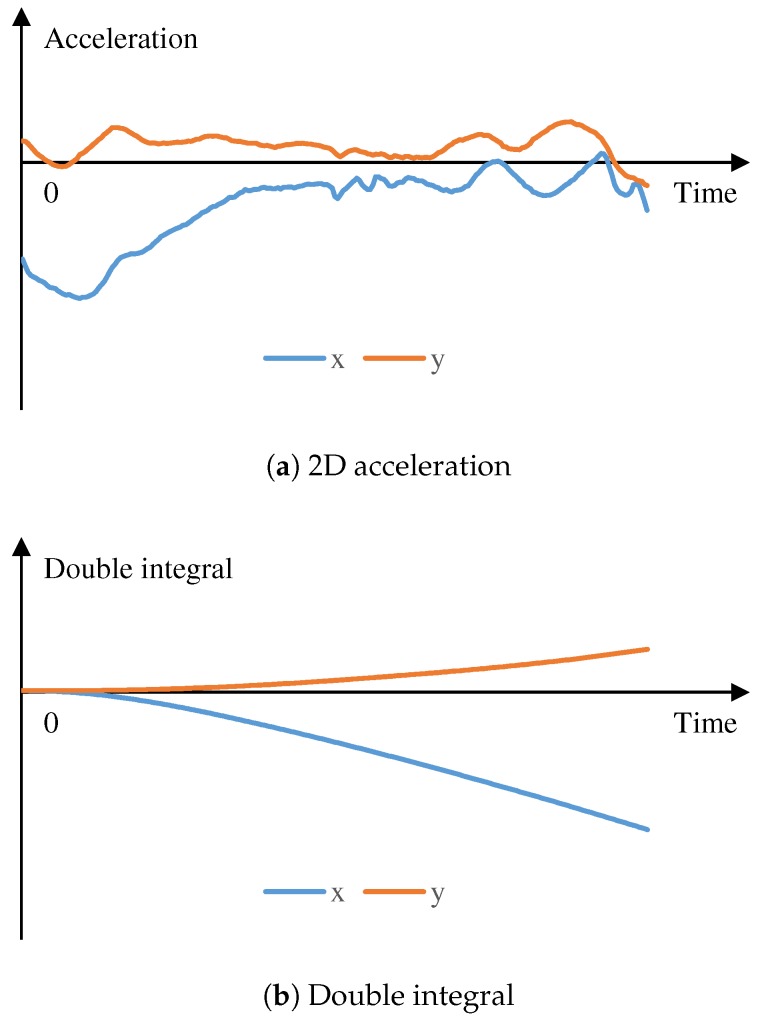
Data of one step.

The motivation of formulating the Equation (1) is as follows. The 2D acceleration data of one step in the xy co-ordinates of the world frame is illustrated in Figure 6a. In this case, the moving-forward direction is along the *x* axis. From the figure, the decelerating phase is obviously more observed than the accelerating phase. We found that this can be a chest-acceleration characteristic. Therefore, the displacement computed by the double integration of the sequential 2D accelerations is opposite the forward direction, as illustrated in Figure 6b. To transform it to correct the direction and length, a user parameter *K* is required.

As a calibration method, *K* is computed for each pedestrian by:

(2) $$K=-\frac{D_{g}}{D_{d}}$$

where $D_{g}$ is a given distance in which the pedestrian is asked to walk straightly, and $D_{d}$ is the total length computed from the double integration of accelerations in one step for all of the steps. To decrease the error on the measurement, we tested five times, and used the mean as the final *K*. For calibration, the longer $D_{g}$ is, the better for accuracy it is. In our experiment, we empirically set $D_{g}$ to 20 m. For a pedestrian whose height is about 170 cm, *K* was normally in the range of [−5.0,−3.0]. After calibration, the error on step length was generally around 10% in our experiments. This error range is used for map matching, as described in Section 3.4.

It should be noted that displacement on the *y* axis was not zero, as illustrated in Figure 6b. Normally, steps are alternately made by the left foot and right foot. Therefore, the upper body tends to alternately lean to the left side and right side. This is the reason why there is side displacement in one step. Since the step made by the left or right foot is usually followed by a step made by the other foot, the overall movement is forward.

For PDR systems based on an upper-body-mounted IMU, it is usually difficult to compute the step direction. Most of the systems require that the step direction is equivalent to the IMU heading. This means that it is assumed that the IMU heading is equivalent to the moving direction. This constraint makes the systems inconvenient to use, and it is not valid when unexpected movements occur. In contrast, our approach does not depend on the actual IMU heading because step displacement is computed from accelerations in the world frame. Therefore, our system has no restriction on step direction with respect to the IMU heading, which is more user-friendly and accurate under several motions.

### 3.4. Map Matching

By applying step detection to the IMU data, the error accumulation caused by the sensor noise can be suppressed compared with the sequential double integration of the accelerations. However, the positioning and heading errors in the world frame still grow over time. To improve accuracy, we integrated the idea of an efficient map-matching algorithm based on particle filtering [24] into our system.

**Figure 7:**
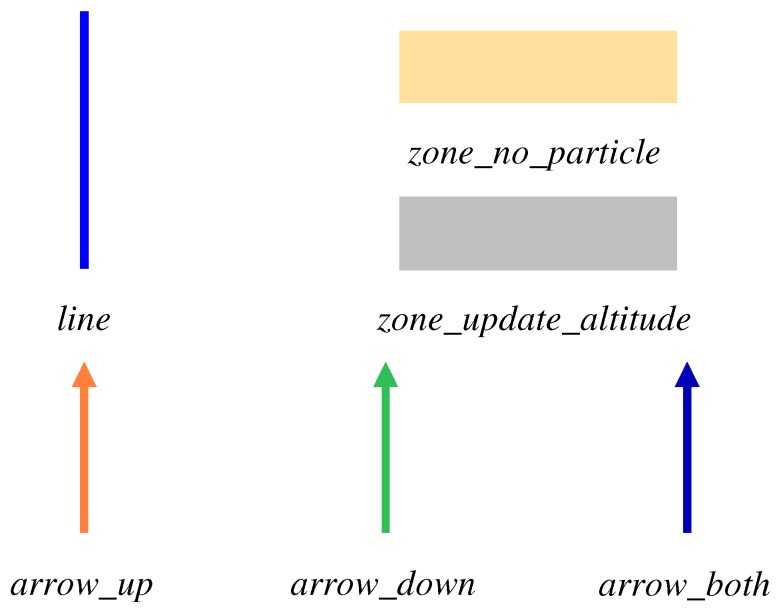
Map components.

As illustrated in Figure 7, we propose to define six components in our map for a multifloor building. line corresponds to a wall that cannot be crossed. In zone_no_particle where pedestrians cannot come into, the existing particles are deleted, and new particles cannot be generated. Only in zone_update_altitude, where altitude can be changed, such as stairs, escalators, and elevators, will the altitude be updated according to the movement. arrow is used for explicitly describing the direction of the possible movement at such locations. arrow_up connects to the upper floor, arrow_down connects to the lower floor, and arrow_both connects to both floors.

**Figure 8:**
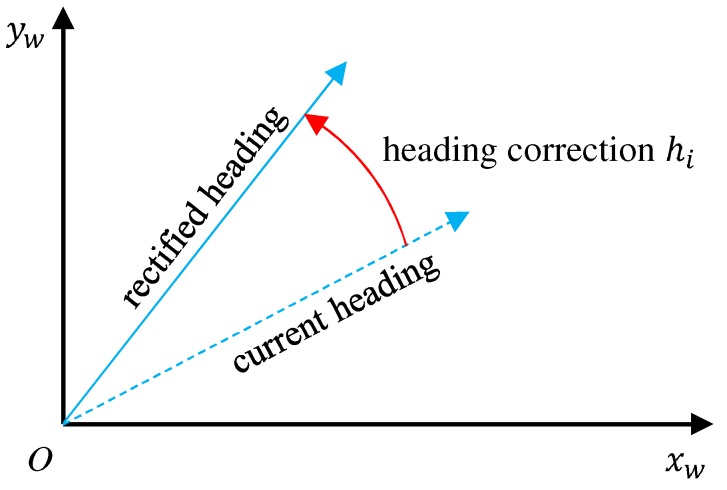
Definition of heading correction $h_{i}$.

In our map-matching algorithm, each particle has three parameters: position $P_{i}$, scale $s_{i}$, and heading correction $h_{i}$ for the *i*-th particle. Scale $s_{i}$ is a scalar value to have error tolerance of the step length, which is randomized from the range [1.0−r,1.0+r], where *r* controls the range. *r* can be determined with the possible error range from preliminary calibration experiments. For instance, r=0.1 when error is up to 10%, as described in Section 3.3. Since the heading in the world frame may drift away from its actual value, as discussed in Section 3.1, we propose to parameterize heading correction $h_{i}$ as a rotation angle to rectify the current heading computed from the IMU orientation into the actual heading in the xy co-ordinate system, as illustrated in Figure 8.

For each particle, position $P_{i}$ is updated by:

(3) $$P_{i}^{t+1}=P_{i}^{t}+R\left(D^{t}\cdot s_{i},h_{i}\right)$$

where $D^{t}$ is the 2D displacement of one current step computed from the IMU, R(X,y) is a function that rotates vector X with angle *y* on the xy co-ordinates. $h_{i}$ and $s_{i}$ are determined and fixed once a new particle is generated. Final position P and heading correction *h* of the pedestrian are finally computed by:

(4) $$\begin{array}{ccc} P & = & \frac{1}{n}\cdot \sum_{i=1}^{n}P_{i} \end{array}$$

(5) $$\begin{array}{ccc} h & = & \frac{1}{n}\cdot \sum_{i=1}^{n}h_{i} \end{array}$$

where *n* is the number of particles.

The procedure of generating new particles is as follows. After updating $P_{i}$ with Equation (3) at every step, our system applies collision detection with the map to the particles. For instance, if the line from $P_{i}^{t}$ to $P_{i}^{t+1}$ intersects with the line, the *i*th particle is deleted, and our system generates new particles for the next step, as described in Algorithm 1 and Table 2. The position of NewParticle is randomized from the circle with center Particle and radius Radius. The heading correction of NewParticle is randomized around final heading correction *h*. The scale of NewParticle is randomized from the range of [1.0−r,1.0+r]. Once NewParticle is generated, its heading correction and scale are fixed.

**Algorithm 1:** Generating new particles.
ParticleNumberMax=256

TryTimeMax=8

Radius=3.0

ParticleNumber=n
while ParticleNumber≤ParticleNumberMax:     Particle=SelectRandomParticle()     TryTime=1     while TryTime≤TryTimeMax:          NewParticle=ProposeNewParticle(Particle,Radius)          if BacktrackingTest(NewParticle)==PASS:               AppendParticle(NewParticle)               ParticleNumber=ParticleNumber+1               break          TryTime=TryTime+1

**Figure 9:**
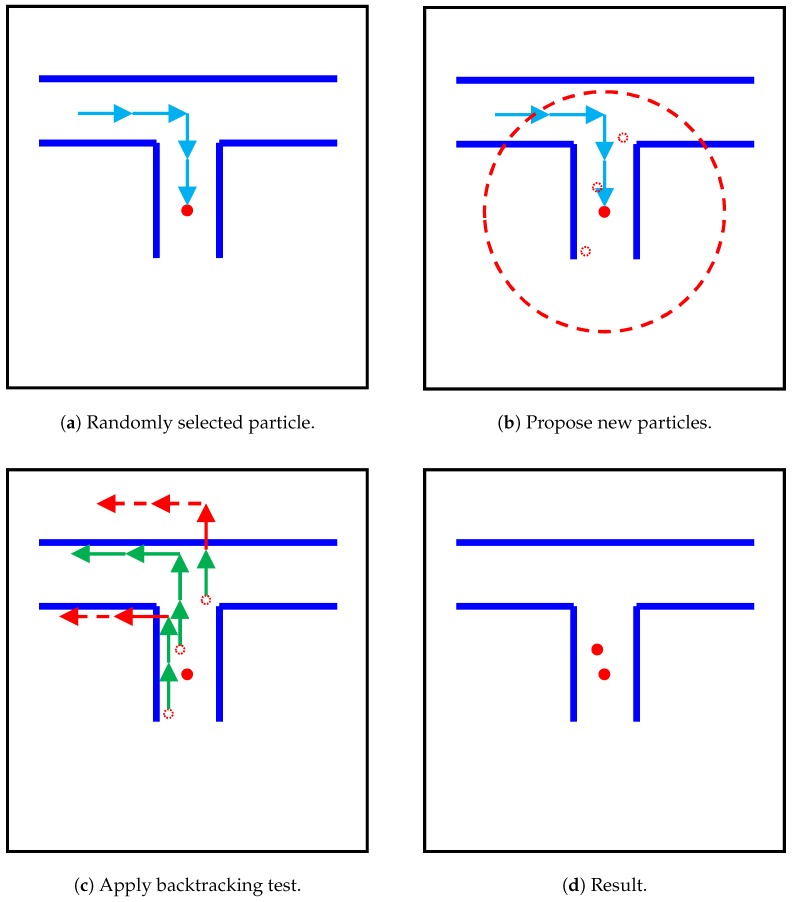
Process of backtracking test. Blue arrows are recent steps.

When generating new particles, the particles may be generated in inappropriate positions. This problem decreases positioning accuracy, and sometimes even crashes the map-matching module. Therefore, inspired by the backtracking particle filter proposed by Widyawan et al. [31], we applied a backtracking test to new particles to select only appropriate particles. In Figure 9, blue arrows correspond to recent steps. First, new particles are randomly proposed around a randomly selected particle. Then, new particles go back recent steps and apply collision detection to the particles, as illustrated in Figure 9c. If a particle hits the wall, it is deleted. As illustrated in Figure 9d, only particles that pass this test are left. By using the backtracking test, most new particles are produced around the correct position. This algorithm largely allows our system to achieve higher accuracy. Moreover, this algorithm could be used when map matching failed to track the user. By applying the backtracking test to randomly selected points around the last estimated position, it is possible to find the correct current position and restore the system. In our experiment, considering real-time processing and accuracy, we used the 32 most recent steps for the backtracking test.

**Figure 10:**
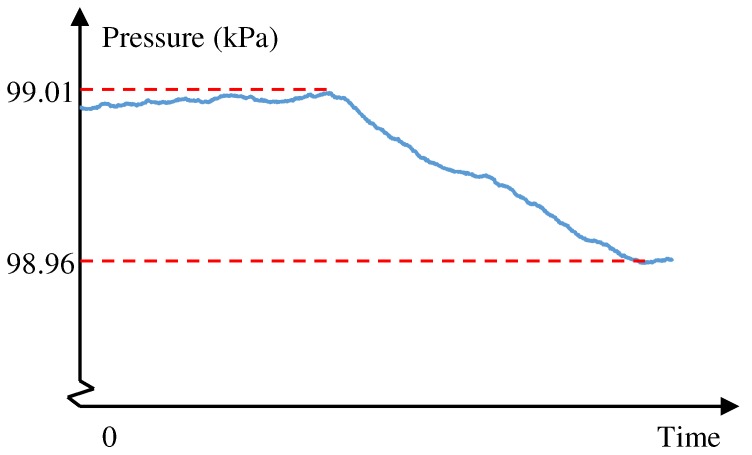
Pressure variations when going upstairs.

For multifloor-building tasks, we use the barometer to update the altitude of the pedestrian. Delta altitude Δa can be computed by:

(6) Δa=Δp·−0.09m/Pa

where Δp is the delta pressure. As illustrated in Figure 10, pressure decreases when going upstairs. However, barometric pressure varies over time even in indoor situations. To correctly update the altitude, we propose to use zone_update_altitude as a component of the map. Only in this zone can the altitude be updated. All arrows are defined in this zone. Since the pedestrian does not stay in this zone for a long time, this approach usually works accurately enough. When delta altitude Δa is closed to the floor height, our system matches the current position to the export of the nearest arrow, and produces new particles. Every time the floor changes, the positioning error of our system can be reset.

### 3.5. Map Editor

**Figure 11:**
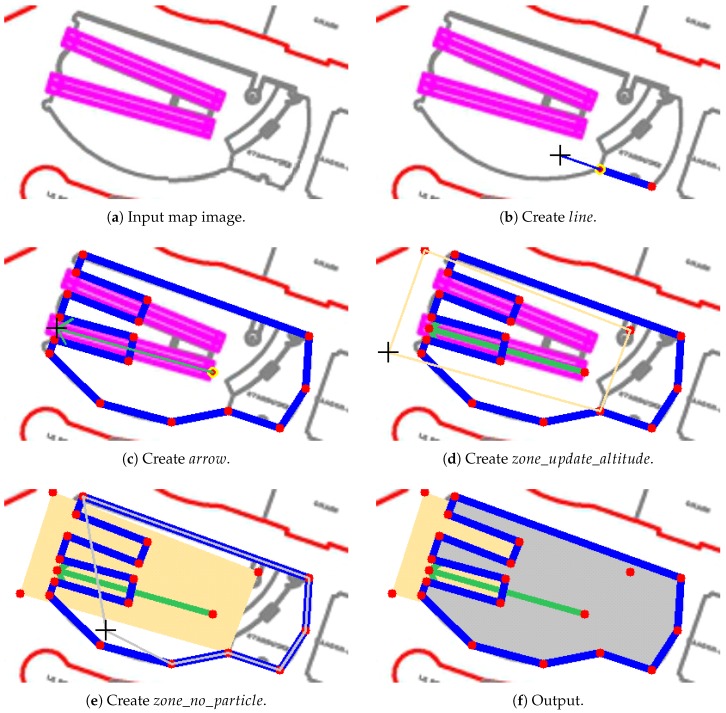
Map-editing process.

To easily generate the map, we developed a map editor so that the map input to our system can be manually edited. The input to the editor is the map images, and the output is a set of files that contain the multifloor-building information. As illustrated in Figure 11, the basic component of our map is a point. By linking the points, we can create any component. Our map editor is available online (https://github.com/rairyuu/PDR-with-Map-Matching).

## 4. Evaluation

In this section, we present the quantitative-experiment results of our system.

### 4.1. Evaluation Criterion

We followed the evaluation procedure used in IPIN 2018 Track 2 as follows. In each experiment, the pedestrian was asked to walk along a given route. Several keypoints, where the positions were measured in order to be known, were set in the route before the experiment. The pedestrian was asked to slightly stop at each keypoint so that the estimated position output by our system was recorded.

Error $e^{i}$ of the *i*-th keypoint is defined by the following equations:

(7) $$e^{i}=D_{xy}\left(P_{g}^{i},P_{e}^{i}\right)+D_{z}\left(P_{g}^{i},P_{e}^{i}\right)$$

(8) $$D_{xy}\left(P_{g}^{i},P_{e}^{i}\right)=\sqrt{{\left(P_{g}^{i}\cdot x-P_{e}^{i}\cdot x\right)}^{2}+{\left(P_{g}^{i}\cdot y-P_{e}^{i}\cdot y\right)}^{2}}$$

(9) $$D_{z}\left(P_{g}^{i},P_{e}^{i}\right)=\left|P_{g}^{i}\cdot z-P_{e}^{i}\cdot z\right|\cdot p_{z}$$

where $P_{g}^{i}$ is the ground-truth position, and $P_{e}^{i}$ is the estimated position. $P^{i}$ has three elements, *x*, *y*, and *z*. *x* and *y* are expressed in meters, whereas element *z* is a scalar that represents the current floor. $p_{z}$ is the penalty on floor error, which is set to 15.

### 4.2. System Initialization

In each experiment, the initial position and heading were given to set up the system as follows.

**Figure 12:**
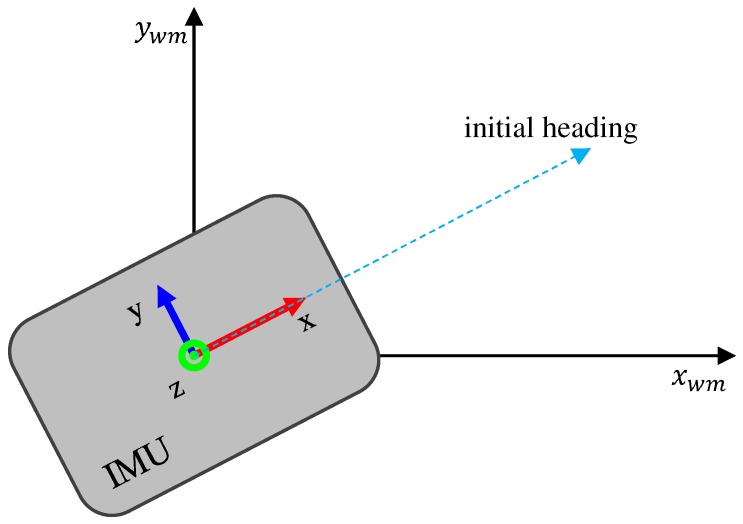
Calibrate IMU pose with initial heading.

1. Open the IMU, launch our system software, and connect the IMU to the computer.
2. Calibrate the IMU pose with the known initial heading in the world frame of the map, as illustrated in Figure 12.
3. Attach the IMU on the pedestrian chest, and run the system.

### 4.3. Lab Experiment

**Figure 13:**
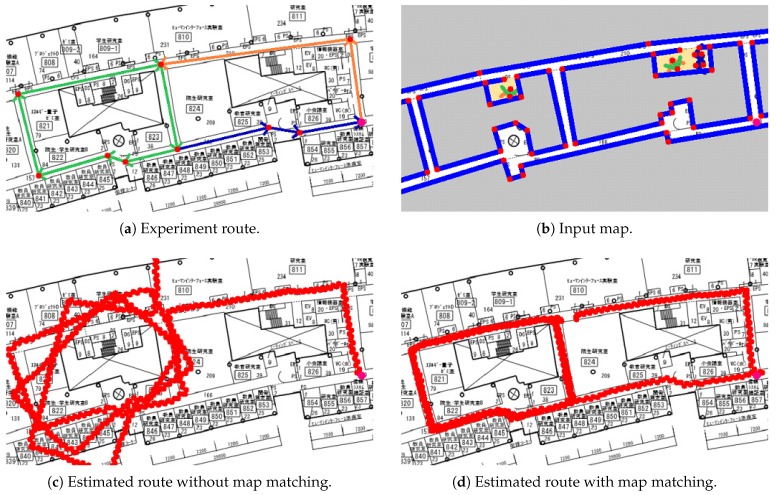
Experiment 1: system evaluation with different configurations.

In the first experiment, we evaluated our system in the campus building with different configurations. As illustrated in Figure 13a, the pedestrian was asked to start at the pink point, go along the orange arrows, walk around the green route for three times, and finally walk back to the starting point along the blue arrows. There was no open-space area in the route, and the width of corridors was about 3 m. In this experiment, we used 256 particles. The travelled distance was 432.22 m. The red points represent the keypoints for the evaluation. Keypoint positions were given by the map of the building.

**Figure 14:**
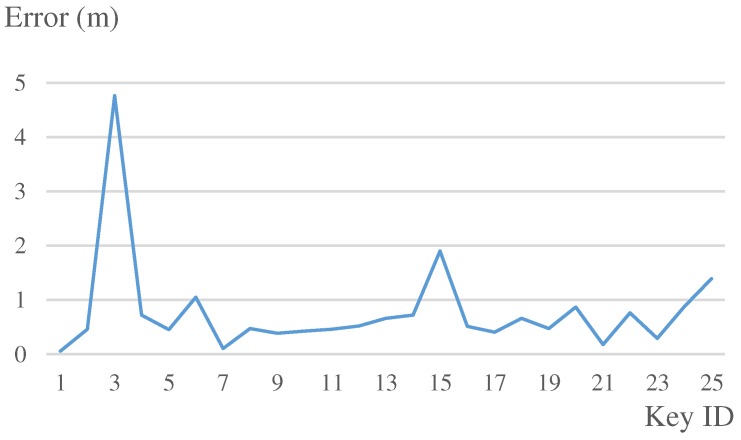
Experiment 1: errors at each keypoint.

The estimated routes of our system are illustrated as Figure 13c,d. At the beginning of the route, the system without map matching was accurate enough. However, the drift on heading was growing over time, as can be seen in the figure. Therefore, the result obviously became worse. In our system with map matching, since the estimated position and heading were calibrated dynamically, the result was much more accurate and stable. The detailed results are illustrated in Figure 14 and Table 3.

### 4.4. IPIN 2018 Competition Experiment

In the second experiment, we evaluated our system by participating in the IPIN 2018 Competition Track 2. The competition was held in a huge shopping mall that contained several open-space areas. The evaluation route crossed three floors, and consisted of indoor and outdoor spaces. In the indoor-space areas, the width range of corridors was from 10 to 20 m. In this experiment, we used 512 particles that were optimized by preliminary experiments. During the competition, the shopping mall was crowded.

**Figure 15:**
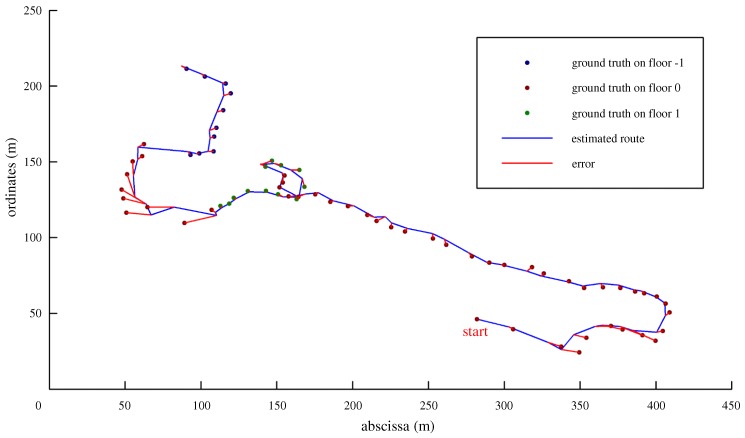
Experiment 2: evaluation in IPIN 2018 Competition Track 2.

**Figure 16:**
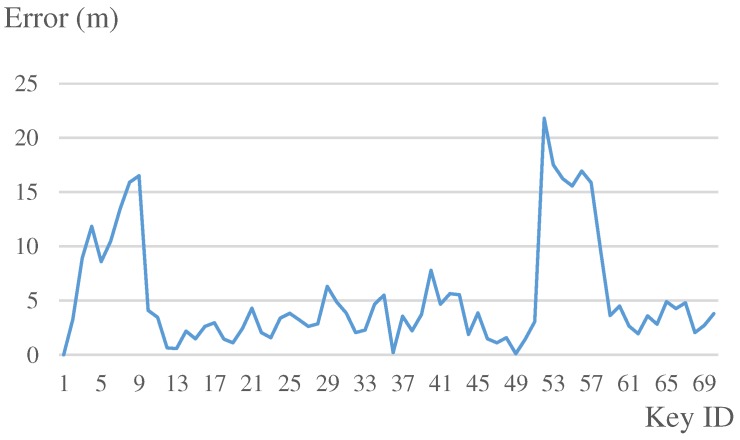
Experiment 2: errors at each keypoint.

The competition route, our estimated result, and the errors are illustrated in Figure 15. The error at each keypoint is illustrated in Figure 16. In outdoor-space areas, since the map matching cannot work, our result was not so accurate. When we went into an indoor area, the result improved, where error was generally less than 5 m. As described in Table 4, the mean error of our system was 5.2 m. The IPIN competition evaluated the 75th percent error. Therefore, our final score was 5.7 m, which was 0.2 m worse than the champion. In the 10 teams who participated in this track, we were awarded second place. The detail of the competition can be found on the IPIN 2018 homepage (http://ipin2018.ifsttar.fr/competition/about/). We also uploaded a video (https://youtu.be/Ti7w-r3dB2E) that provides a more detailed result and shows how our map matching worked in the competition.

## 5. Conclusions

In this paper, we proposed a novel indoor positioning system with map matching. In our system, the IMU is mounted on the chest where movement is simplest and most stable. To reduce error accumulation on position and heading, we designed an efficient map-matching algorithm based on particle filtering. Different from most existing algorithms, our map-matching algorithm was designed for 3D navigation, which can be used in multifloor buildings. With carefully designed components (wall, stair, escalator, elevator), our map can clearly represent building-floor information. With the proposed algorithm implemented, our navigation system was awarded second place in the IPIN 2018 Competition Track 2, achieving a mean error of 5.2 m after 800 m of walking. In our future work, we are improving the accuracy of the step-length estimation. As mentioned in our motivation, we are also designing a body suit to support the indoor work.

## Figures and Tables

**Table 1:** NGIMU specifications.

	Range	Resolution	Sampling Rate
Accelerometer	±16 g	490 μg	400 Hz
Gyroscope	$\pm 2000^{\circ }$/s	$0.06^{\circ }$/s	400 Hz
Barometer	30∼110 kPa	0.18 Pa	25 Hz
Size		56 × 39 × 18 mm	
Weight		46 g	

**Table 2:** Definition of variables and functions in Algorithm 1.

ParticleNumberMax	Maximal number of particles in our system.
ParticleNumber	Number of current existing particles.
*n*	Number of existing particles before generating new particles.
TryTimeMax	Maximal trying time on proposing a new particle.
TryTime	Counter of trying time on proposing a new particle.
Particle	Randomly selected particle.
Radius	Second input parameter of function ProposeNewParticle.
SelectRandomParticle()	Randomly select one particle from existing particles and return it.
ProposeNewParticle(_particle,_radius)	Propose a new particle around _particle and return it.
BacktrackingTest(_particle)	Apply backtracking test to _particle. Return PASS if _particle passed the test.
AppendParticle(_particle)	Append _particle to existing particles.

**Table 3:** Experiment 1: error distribution.

Travelled distance: 432.22 m	
Total number of keypoints: 25	
	Our system
Mean	0.78 m
Median	0.51 m
75th percent	0.76 m
Standard deviation	0.92 m

**Table 4:** Experiment 2: error distribution.

Travelled distance: 792.49 m	
Keypoint number: 70	
	Our system
Mean	5.2 m
Median	3.6 m
75th percent	5.7 m
Standard deviation	5.0 m
